# Educational benefits for nurses and nursing students of the dementia supporter training program in Japan

**DOI:** 10.1371/journal.pone.0200586

**Published:** 2018-07-19

**Authors:** Yukihisa Matsuda, Reiko Hashimoto, Sachiko Takemoto, Yuka Yoshioka, Takashi Uehara, Yasuhiro Kawasaki

**Affiliations:** 1 Faculty of Pharmacy and Pharmaceutical Sciences, Fukuyama University, Hiroshima, Japan; 2 Department of Neuropsychiatry, Kanazawa Medical University, Ishikawa, Japan; Istituto Di Ricerche Farmacologiche Mario Negri, ITALY

## Abstract

In Japan, which is thought to be a rapidly growing super-aging society, a national campaign named “*the Dementia Supporter Caravan*” has been deployed. The aim of this study was to assess the educational benefits of the dementia supporter training program for nurses and nursing students. We conducted dementia supporter training, and measured knowledge and attitudes regarding people with dementia as educational benefits pre- and post-training. Data sets of 134 nursing students and 63 nurses were analyzed. The results indicated that the two groups gained knowledge, understanding, and the confidence to care for people with dementia after attending the dementia supporter training program. Moreover, the two groups derived different benefits from the program. Nursing students gained substantial knowledge and learnt the importance of early detection and treatment, to levels similar to those of nurses prior to training. The training program reduced the difficulties of nurses to interact with and care for people with dementia. We can conclude that the dementia supporter training program has considerable educational benefits for nurses and nursing students.

## Introduction

The proportion of older people is rapidly increasing in most global populations, and this is becoming a major health issue around the world [1]. Japan, which is considered a growing super-ageing society [2], has a population of 34.4 million people aged ≥65years, accounting for more than a quarter of the country’s entire population in 2016 [3]. Increases in the number of older people require social, economic, and medical changes, including measures of dementia. Dementia is a clinical syndrome that involves progressive deterioration of intellectual function, such as memory, executive functioning, and language [4]. Dementia covers a wide range of symptoms of disease and has subtypes, Alzheimer’s disease [5], vascular dementia [6], mixed dementia [7], dementia with Lewy bodies [8], and frontotemporal dementia [9].

To address these, the Japanese Ministry of Health, Labour and Welfare established the “Five-Year Plan for Promotion of Measures Against Dementia (Orange Plan)” in September 2012 [10] and the “Comprehensive Strategy to Accelerate Dementia Measures (New Orange Plan)” in January 2015 [11]. The New Orange Plan advances awareness-raising and facilitates an understanding of dementia, and promotes dementia-friendly communities [12]. The concept of dementia-friendly communities is to normalize dementia in society [13]. Dementia-friendly communities (societies) have “inclusive and accessible community environments that optimize opportunities for health, participation and security for all people, in order to ensure quality of life and dignity for people with dementia, their caregivers and families” [14]. To realize such dementia-friendly communities, nationwide campaigns and community-based activities have been conducted at over 40 countries [15].

A dementia awareness-training program known as *the Dementia Supporter Caravan* in Japan is a national campaign aligned with the New Orange Plan. The Dementia Supporter Caravan is a campaign to encourage early detection and treatment while reducing prejudice and gaining awareness. It has a three-tier approach. Firstly, medical professionals specializing in dementia train people who are engaging medical services, such as care managers, nursing staff, doctors, and volunteer leaders. People who have been trained by medical professionals are called *caravan mates*. Secondly, caravan mates provide educational programs and training to local residents, students, caretakers, hospital workers, and so on. People who have been trained by caravan mates are called as *dementia supporters* (or *“Ninchisho” supporters*). Thirdly, dementia supporters support older people, people with dementia, and their families in everyday life [16].

Though dementia supporters have shallower knowledge compared with caravan mates, the highest priority on the dementia supporter caravan is to spread dementia-friendly minds throughout Japan. Thus, this broad-based approach is the best way to construct dementia-friendly communities. This “train the trainer” approach makes much number of supporters who have good understanding about people with dementia, this situation is characterized as a “caravan”.

Those awareness-training programs as the dementia supporter caravan were held by municipalities, schools, and companies. Participants were recruited by announcements using local papers, postings, flyers, web sites and so on. To date, about 242,000 *dementia supporter training programs* which train participants to become dementia supporters have been held by caravan mates, and the number of dementia supporters has reached eight million [17]. The Dementia Supporter Caravan is becoming a nationwide campaign, but only a few studies have examined the educational benefits of the Dementia Supporter Caravan (e. g., [18]). Measuring the educational benefits is thought to be an important step in providing evidence on the need to enhance and expand on the Dementia Supporter Caravan.

The aim of this study was to measure the educational benefits of the dementia supporter training program for nurses and nursing students, and to examine any differences in the educational benefits to the two groups. We focused on nurses and nursing students as targets of the dementia supporter training program. Nurses have been engaging in clinical practice with various patients, including patients with dementia and older hospital patients. On the other hand, nursing students have had fewer opportunities to engage in such activities. Previous studies have shown that clinical experience for nursing students led much knowledge and improved attitudes toward people with Alzheimer’s disease [19], toward people with dementia [20], and higher level of knowledge about dementia was associated with previous experience in caring for people with dementia [21]. Thus, the clinical experience is an important factor for knowledge and attitude toward people with dementia. We hypothesized that the educational benefits of the dementia supporter training program would especially favor nursing students. Nursing students have less clinical experience, so it is considered that they have less knowledge and confidence to interact with people with dementia and aged people before attending the dementia supporter training program. Previous study also showed that higher level of knowledge about dementia was also associated with experiences in education on dementia [21]. From this finding, it is hypothesized that nursing students acquire much educational benefits of dementia after the dementia supporter training program. To assess educational benefits, we conducted a knowledge test and an awareness survey related to dementia before and after the dementia supporter training program.

## Materials and methods

### Participants

Participants for this study consisted of 150 nursing students (12 male, 138 female, mean age ± SD: 19.33 ± 0.57 years) and 72 nurses (5 male, 67 female, mean age ± SD: 43.14 ± 12.87 years). All nursing students were second-year undergraduate students at the school of nursing at Kanazawa Medical University. All nurses worked at Kanazawa Medical University Hospital. This study was performed according to the World Medical Association’s Declaration of Helsinki and approved by the Research Ethical Committee of Kanazawa Medical University. The purpose and procedures of this study were explained to participants. Only the data from participants who had provided informed consent were used. Those who did not give informed consent and wanted to participate in the training program were allowed to do so without completing any measures.

### The dementia supporter training program

Six trainers were trained on how to conduct the dementia supporter training program, which is aligned to an official textbook and PowerPoint slides created by the association of the Dementia Supporter Caravan. Trainees were supervised by two specialists affiliated with the Dementia Supporter Caravan. Three trainers delivered the training program to nursing students, and the other three to nurses. Each program took approximately 90 minutes.

The two training programs were conducted on different days. The training program consisted of the following: 1) explanations of common types of dementia are Alzheimer’s disease, dementia with Lewy bodies, frontemporal dementia, vascular dementia, and others, 2) core symptoms and behavioral and psychological symptoms (BPSD), 3) the necessity of early detection and treatment, 4) prevention of dementia, 5) how to provide support, 6) how to correspond, 7) attitudes towards people with dementia, 8) about dementia supporters.

Prior to the training program (pre-training period), a three-part questionnaire including demographic questions, a dementia knowledge test based on the Dementia Supporter Caravan text (the DKT-SC), and an Awareness Questionnaire, was distributed to participants. Participants were required to complete to the questionnaire. The questionnaire took approximately 10 minutes to complete. After completion of the program (post-training period), participants were required to complete the DKT-SC and the Awareness Questionnaire.

### Measures

#### The DKT-SC

The DKT-SC consists of 18 items examining knowledge about dementia (Table 1). Three psychologists and two medical doctors chose 80 features of dementia from the official textbook, and then eliminated duplicated and/or ambiguous features. They then assessed the importance of each feature as an item; 18 items were selected. The DKT-SC was two-alternative forced-choice test, with participants required to judge whether each item was correct or incorrect.

**Table 1:** The dementia knowledge test.

Item #	Item	Correct Answer
1	Behavioral and psychological symptoms of dementia (BPSD) are core symptoms of dementia.	False
2	Because the progression of Alzheimer’s disease cannot be stopped, the timing of when to go to medical institutions is not an issue.	False
3	When encounting people with dementia, “do not be surprised”, “do not rush”, “do not allow them to exercise judgement”.	False
4	The onset of executive dysfunction makes it impossible to mentally plan and make arrangements.	True
5	Regarding the memory of people with dementia, living skills acquired in the past are retained.	True
6	Delusion is not a symptom of dementia.	False
7	High blood pressure, hyperlipidemia, obesity, and lifestyle-related diseases are not related to dementia.	False
8	In the diagnosis of dementia, imaging tests such as CT scans are not very effective.	False
9	Memory impairment in dementia leads to forgetting having eaten meals.	True
10	Disorientation is a symptom of face recognition impairment.	False
11	The most important thing in dealing with people with dementia is to watch over them.	True
12	When starting to talk to people with dementia, it is better to address them from the front.	True
13	If you have dementia, understanding of one’s situation occurs at a relatively early stage.	False
14	Disorientation in people with dementia appears early, in parallel with memory impairment.	True
15	If you have dementia, you cannot handle an overlap between two or more things.	True
16	Alcoholism is also one of the factors that can cause dementia.	True
17	As dementia progresses, the symptoms progress from “delusion of theft” to “memory disorder”.	False
18	When dementia progresses, micturition and bowel movements become difficult to feel.	True

#### The Awareness Questionnaire

The questionnaire consists of 17 items (Table 2). Items 1 to 8 are based on a questionnaire previously used by Saito and Tsuchiya [18]. The Awareness Questionnaire covered the following issues: interest in the Dementia Supporter Caravan (items 1 and 5), interest in dementia (item 6), relationship with patients who have dementia or older people (item 2 to 4), difficulty interacting with dementia (item 8), confidence in care provision (item 9), and understanding of dementia (items 7 and 10 to 17). In items 1 to 4, the number of alternatives varied according to content. Items 5 to 17 were measured on a five-point Likert scale (e. g., “strongly disagree” to “strongly agree”).

**Table 2:** The Awareness Questionnaire of dementia.

Item #	Item and alternative
1	Do you know about the Dementia Supporter Caravan activity?
	1. I know about it
	2. I have heard about it
	3. I have never heard of it
	4. I know about it; I have the Orange Ring.
2	Are there any people with dementia who are close to you?
	1. Yes
	2. No
3	Have you lived with people older than 65 years of age?
	1. I have lived with older people with dementia
	2. I have lived with older people who do not have dementia
	3. No
4	Have you ever interacted with people with dementia?
	1. No
	2. For several times
	3. From several weeks to about a year
	4. For more than a few years
5	How interested are you in the Dementia Supporter Caravan activity?
	1. Not at all interested—5. Very interested
6	How interested are you in dementia?
	1. Not at all interested—5. Very interested
7	Do you agree or disagree that dementia requires early diagnosis and early treatment?
	1. Strongly disagree—5. Strongly agree
8	How difficult is it to have relatives with people with dementia and/or to be involved in their care?
	1. Very difficult—5. Not difficult at all
9	How confident are you about your ability to care for people with dementia?
	1. Not at all confident—5. Very confident
10	Do you agree or disagree that dementia is a disease of the brain?
	1. Strongly disagree—5. Strongly agree
11	Do you agree or disagree that people with dementia cannot understand even if they are spoken to?
	1. Strongly disagree—5. Strongly agree
12	Do you agree or disagree that there is a possibility that anyone could develop dementia?
	1. Strongly disagree—5. Strongly agree
13	Do you agree or disagree that there are drugs that delay the progress of dementia?
	1. Strongly disagree—5. Strongly agree
14	Do you agree or disagree that, even if you have dementia, you could live a normal life?
	1. Strongly disagree—5. Strongly agree
15	Do you agree or disagree that people with dementia do not have insight into their illness?
	1. Strongly disagree—5. Strongly agree
16	Do you agree or disagree that it is necessary to have adequate knowledge and understanding of dementia?
	1. Strongly disagree—5. Strongly agree
17	Do you agree or disagree that it is necessary to support people with dementia and their families?
	1. Strongly disagree—5. Strongly agree

### Statistical analysis

The characteristics of the nursing-student group and the nurse group were presented in the form of numbers and percentages. In the analyses of accuracy (ACCs) of the DKT-SC, a two-way mixed analysis of variance (ANOVA) was performed with group (nursing students and nurses) as the between-group factor and period (pre-training and post-training) as the within-group factor. In the analyses of Awareness Questionnaire, Analyses of scores on items 5 to 17 were conducted separately. Two-way mixed ANOVAs were performed with group and period. Analyses were performed using IBM SPSS Statistics Standard version 22 (IBM Japan; Tokyo, Japan) and ANOVA4 (http://www.hju.ac.jp/~kiriki/anova4). All reported *p*-values were two tailed, with *p* < 0.05 indicating significance. Effect sizes for simple effects of ANOVA were calculated as Cohen’s *d* (*d*).

## Results

There were no dropouts between pre-training period and post-training period. Sixteen participants in the nursing-students group (10.7%) and nine in the nurses group (12.5%) were excluded due to incomplete data. Data by 134 nursing students and 63 nurses were used in the analysis. Demographic data and the results for items 1 to 4 are shown in Table 3.

**Table 3:** Characteristics of participants in this study.

	Nursing student	Nurse
Age (mean ± SD)	19.31 ± 0.57	42.63 ± 13.14
Sex (*n*, %)		
Males	11 (8.2)	5 (7.9)
Females	123 (91.8)	58 (92.1)
Awareness Questionnaire (*n*, %)		
Item 1: Do you know the Dementia Supporter Caravan activity?		
1. I know	13 (9.70)	9 (14.29)
2. I have heard	15 (11.19)	15 (23.81)
3. I have never heard	92 (68.66)	37 (58.73)
4. I know; I have the Orange Ring.	14 (10.45)	2 (3.17)
Item 2: Are there any people with dementia who are close to you?		
1. Yes	26 (19.40)	22 (34.92)
2. No	108 (80.60)	41 (65.08)
Item 3: Have you lived with older people aged >65 years?		
1. I have lived with older people with dementia	14 (10.45)	17 (26.98)
2. I have lived with older people who do not have dementia	49 (36.57)	29 (46.03)
3. No	71 (52.99)	17 (26.98)
Item 4: Have you ever had interacted with people with dementia?		
1. No	77 (57.46)	1 (1.59)
2. Several times	30 (22.39)	14 (22.22)
3. Several weeks to about a year	5 (3.73)	17 (26.98)
4. More than a few years	22 (16.42)	31 (49.21)

### ACCs of the dementia knowledge test

**Fig 1:**
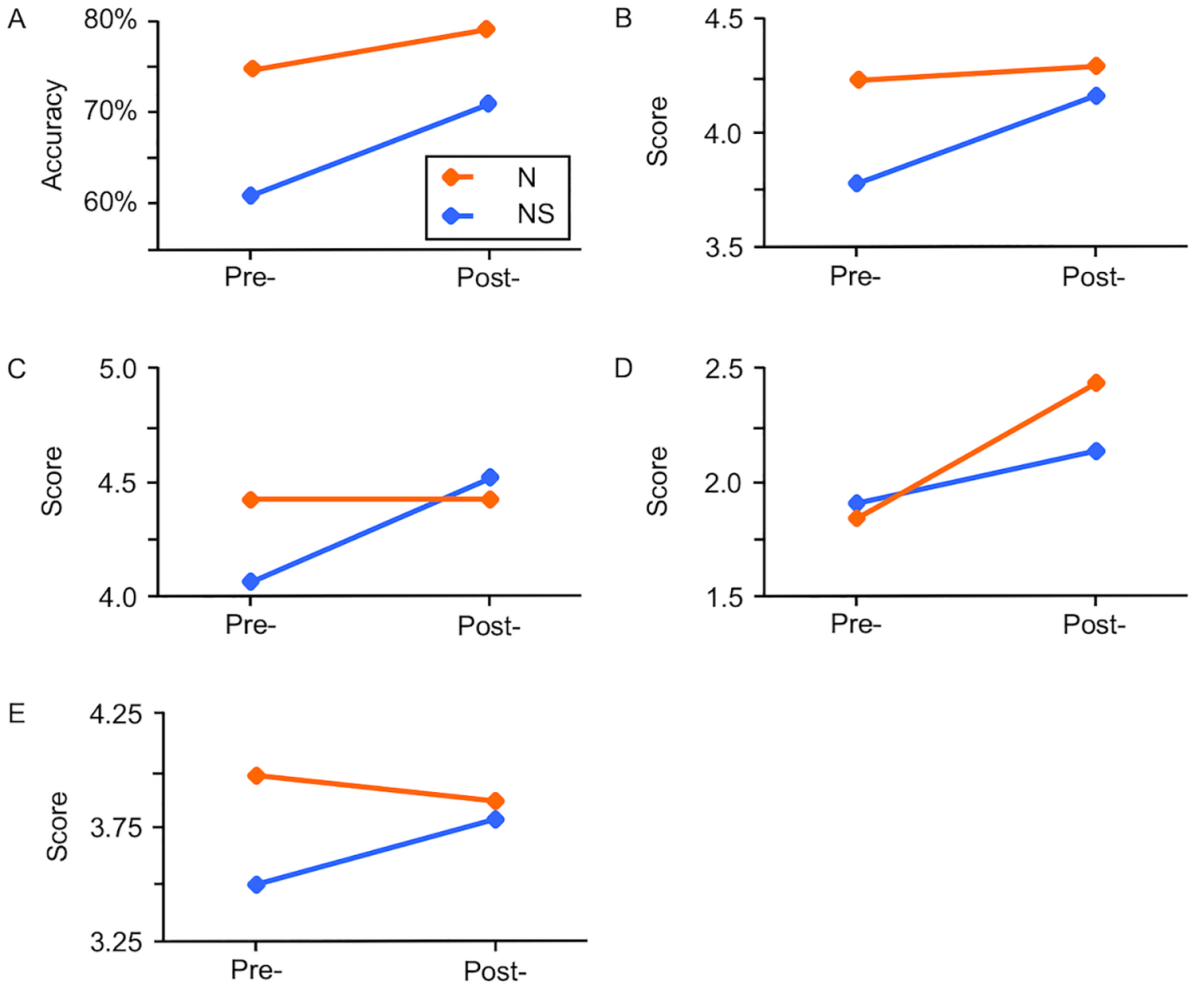
Data plot at the pre-training and post-training periods. Mean ACCs of the dementia knowledge test were shown in Panel A. Mean scores on items 6, 7, 8, and 13 of the Awareness Questionnaire were shown in Panel B, C, D, and E, respectively. Orange line represents the nurse group. Blue line represents the nursing-student group.

The results of the main effect of group, period, and the interaction between the two factors are shown in Table 4. Simple effects of two-way interactions were significant (Fig 1, Panel A); the ACC at the post-training period was higher than that at the pre-training period in the nursing-student group [*F*(1, 195) = 62.99, *p* < 0.001, *d* = 0.91] and in the nurse group [*F*(1, 195) = 12.51, *p* < 0.001, *d* = 0.42]. The ACCs of the two groups at each period also showed significant differences. The ACC of the nurse group was higher than that of the nursing-student group at pre-training [*F*(1, 390) = 56.25, *p* < 0.001, *d* = 1.11] and post-training [*F*(1, 390) = 17.22, *p* < 0.001, *d* = 0.66].

**Table 4:** Mean data and the results of analysis of variance.

	Nursing student		Nurse		Results of ANOVA (*F*)		
	Pre	Post	Pre	Post	Group	Period	Interaction
Knowledge test (%, SD)							
	61.32 (11.73)	71.81 (11.23)	74.34 (11.87)	79.01 (10.29)	47.78****	65.82****	9.68***
Awareness Questionnaire (score, SD)							
5	3.28 (0.88)	3.96 (0.75)	3.48 (1.01)	4.09 (0.75)	2.29	95.16****	0.25
6	3.78 (0.78)	4.16 (0.68)	4.24 (0.73)	4.30 (0.61)	9.90***	17.46****	9.02***
7	4.06 (1.52)	4.53 (1.18)	4.43 (1.18)	4.43 (1.14)	0.66	4.43*	4.43*
8	1.92 (0.89)	2.14 (1.01)	1.84 (0.82)	2.44 (0.91)	0.84	34.78****	7.31**
9	1.89 (0.94)	2.16 (0.87)	2.33 (0.98)	2.69 (0.87)	15.44****	23.81****	0.55
10	4.09 (1.05)	4.22 (1.04)	4.14 (1.07)	4.32 (0.89)	0.31	3.28	0.06
11	2.52 (1.08)	2.35 (1.19)	2.52 (1.13)	2.09 (1.09)	0.67	10.88***	2.17
12	4.68 (0.83)	4.63 (0.78)	4.67 (0.54)	4.57 (0.87)	0.14	1.14	0.15
13	3.54 (1.34)	4.08 (1.20)	4.48 (0.69)	4.27 (0.99)	13.14****	2.45	12.09****
14	3.96 (1.02)	4.23 (0.94)	4.32 (0.71)	4.54 (0.59)	6.38*	18.42****	0.84
15	3.60 (1.22)	3.08 (1.43)	3.18 (1.33)	2.69 (1.44)	5.68*	17.80****	0.05
16	4.79 (0.62)	4.89 (0.43)	4.87 (0.38)	4.84 (0.44)	0.04	1.04	3.65
17	4.79 (0.61)	4.87 (0.49)	4.91 (0.29)	4.86 (0.43)	0.57	0.18	3.66

*p < 0.05,

**p < 0.01,

***p < 0.005,

****p < 0.001.

### Scores on the Awareness Questionnaire of dementia

In the analyses of items 6, 9, and 13 to 15, the main effects of group were significant. The nurse group obtained higher scores than did the nursing-student group for items 6, 9, 13, and 14. The nurse group obtained lower scores than did the nursing-student group for item 15.

For items 5 to 9, 11, 14, and 15, the main effects of period were significant. Scores for items 5 to 9 and 14 were higher at the post-training period, compared to the pre-training period. For items 11 and 15, scores at the post-training period were lower than those at the pre-training period.

Simple effects of two-way interactions were significant for items 6 to 8, and 13. The nursing-student group showed higher scores at the post-training period than that at the pre-training period in the analysis for item 6 [Fig 1, Panel B; *F*(1, 195) = 25.79, *p* < 0.001, *d* = 0.52], item 7 [Fig 1, Panel C; *F*(1, 195) = 8.85, *p* < 0.01, *d* = 0.35], item 8 [Fig 1, Panel D; *F*(1, 195) = 5.09, *p* < 0.05, *d* = 0.23], and item 13 [Fig 1, Panel E; *F*(1, 195) = 18.54, *p* < 0.001, *d* = 0.43]. In the analysis for item 13, the nurse group also showed increasing scores at the post-training period [Fig 1, Panel E; *F*(1, 195) = 36.99, *p* < 0.001, *d* = 0.25].

At the pre-training period, nurse group’s mean score was higher than that of the nursing-student group in the analysis for item 6 [Fig 1, Panel B; *F*(1, 390) = 17.80, *p* < 0.001, *d* = 0.60] and item 13[Fig 1, Panel E; *F*(1, 390) = 27.41, *p* < 0.001, *d* = 0.80]. At the post-training period, nurse group’s mean score was higher than that of the nursing-student group in the analysis for item 8 [Fig 1, Panel D; *F*(1, 390) = 4.55, *p* < 0.05, *d* = 0.31].

## Discussion

### Overall benefits of the dementia supporter training program

Despite the results of the DKT-SC and items 6 to 8 of Awareness Questionnaire, given significant interactions between group and period, we confirmed the educational benefits of the training program, as shown by the significant differences in the main effects of period for items 5, 9, 11, 14, and 15 of Awareness Questionnaire. These results suggested that both the nursing-student group and the nurse group developed more interest in the Dementia Supporter Caravan activity (item 5), confidence regarding people with dementia (item 9), and understanding of people with dementia (items 11, 14, and 15) after participation in the training program.

### Differences between nursing students and nurses

We confirmed group differences between the nursing-student group and the nurse group, as shown by the significant differences of the main effects of group in the results of the DKT-SC, items 6, 9, and 13 to 15 of Awareness Questionnaire. The nurse group had substantial knowledge of dementia, as shown by ACCs on the DKT-SC, confidence regarding people with dementia (item 9), and understanding of people with dementia (items 14 and 15). The nurse group had more opportunities to interact with people with dementia than did those in the nursing-student group, as shown in the results for items 3 and 4. The nurse group also had longer interactions with people with dementia, as shown by the results for item 5. These differences might be due to the nurse group having more knowledge, confidence, and understanding, compared to the nursing-student group.

### Influence of group differences on benefits

As we expected, the amount of educational benefits differed between the two groups. There were significant interactions between group and period in the results of the DKT-SC, items 6 to 8, and 13 of Awareness Questionnaire (Fig 1). The ACC of the DKT-SC increased at the post-training period for the nurse group; the increase reached significance, although to a somewhat marginal extent. On the other hand, in the nursing-student group, the ACC clearly increased at the post-training period (Fig 1, Panel A). No educational benefit was shown for the nurse group, whereas substantial benefits were shown for the nursing-student group in the results of items 6, 7, and 13 (Fig 1, Panels B, C, and E). The nurse group obtained higher scores even at the pre-training period for all of the assessments. These results showed that the nurse group had enough knowledge, interest, and understanding of people with dementia. Although the nursing-student group obtained lower scores than those of the nurse group at the pre-training period, their scores increased to levels similar to those of the nurse group at the post-training period. Those results indicated that the nursing-student group got considerable educational benefits from the training program.

The nurse group showed decrease in the difficulty to interact with or care for people with dementia as shown by the *increase* in the mean score for item 8 (Fig 1D). Only this result showed more benefits for the nurse group, compared to the nursing-student group. A possible explanation for this might be that the nurse group had been exposed to interact with and caring for patients in hospital or daily life, which enables them to easily anticipate the utility of the educational benefits. This difference in levels of familiarity with people with dementia might have resulted in the scores obtained for item 8.

### Limitations and further study

This study has several limitations. At present, there are many tools which measure acceptable reliability and validity; the Alzheimer’s Disease Knowledge Scale [22], the Knowledge of Memory Loss and Care Test [23], the Dementia Quiz [24], the Dementia Knowledge Assessment Tool [25], and the Dementia Knowledge Assessment Scale [26]. In this study, our main aim was to address the educational benefits of the dementia supporter training program specifically. Thus, we developed the tools that aligned with contents of the dementia supporter training program and official textbook. The original tools also enable reduced redundancies and time cost. On the other hand, those tools have not yet been checked for validity and reliability. Further studies using measures of acceptable reliability and validity are needed to confirm the generality of the findings in this study.

All training programs had conducted using the same official textbook and PowerPoint slides created by the association of the Dementia Supporter Caravan. In particular, caravan mates were trained to conduct the same programs aligned with the same PowerPoint slides and the same time schedule. Thus, all training programs were considered to be identical. But, all caravan mates had individual speaking styles such as speed and tone of voice. The training programs for nurses and nursing students were conducted separately, thus those factors were not controlled for. It is possible that those factors might influence differently for the educational benefits of the nurse group and the nursing students group.

We obtained participants’ characteristics through items 1 to 4 of the Awareness Questionnaire. For example, it is sensible to compare the educational benefits of participants who had lived with older people and participants who had not. This comparison or that entailing other combinations requires a larger sample size owing to the resultant higher number of groups to compare; however, there is not enough sample size in this study.

It is unclear as to whether the influence of the supporter training program on educational benefits extended to the clinical situation. We conducted the post-test shortly after completion of the training program, with participants not yet having had the opportunity to utilize what they had learnt from the training program. In the previous study using meta-analysis, didactic sessions alone are unlikely to change professional practice in health care settings, but interactive workshops can result in moderately large changes in professional practice [27]. Other studies confirmed that clinical practice improved nursing students’ knowledge of dementia [20]; thus, the knowledge of dementia, as obtained from the training program, and clinical practice might mutually enhance one another.

Recently, web-based learning courses and support for caregivers (e-learning) are actively being delivered [28–30]. E-learning is easily accessible and can provide more opportunities for one to access information about dementia and reduce program implementers’ efforts, compared to face-to-face lectures and training. E-learning also has the benefits of long-term education and relationships between experts and people requiring information and consultation, such as caregivers. Hattink et al. [30] conducted e-learning for two to four months, they confirmed positive effects on attitudes towards dementia and on the person-centered care approach that recognizes the individuality of the patients in relation to the attitudes and care practices that surround them [31]. The Dementia Supporter Caravan is a lecture-style activity entailing the training of dozens of people by several lecturers. Moreover, each training program is typically provided only once or a few times to a given group. This does not facilitate easy access and long-term education. In terms of education duration, Kang et al. [32] conducted a 3-month educational program for nurses on care for older adults with cognitive impairment. That long-term education showed a positive impact on nurses’ knowledge of cognitive impairment and attitudes, also increasing nurses’ initial efforts to involve family caregivers. The next step for the Dementia Supporter Caravan is the development of an easily accessible system such as e-learning and examination of the benefits of long-term education.

### Conclusion

This study showed evidence of the educational benefits of the dementia supporter training program. The current results suggest that the training program imparted substantial educational benefits to nursing students and nurses. The two groups gained considerable knowledge, understanding, and the confidence to care for people with dementia after attending the training program. Moreover, the benefits to the two groups differed. Nursing students, but not nurses, gained considerable knowledge and tended to recognize the necessity of early detection and treatment. Among nurses, but not nursing students, the training program led to a decline in the difficulty with which participants interacted to and cared for people with dementia.
