# Correction: Educational benefits for nurses and nursing students of the dementia supporter training program in Japan

**DOI:** 10.1371/journal.pone.0203232

**Published:** 2018-08-24

**Authors:** The PLOS ONE Staff

The Funding section for this paper is incorrect. The publisher apologizes for the error. The correct funding information is as follows: This work was supported by the Grant for Specially Promoted Research from Kanazawa Medical University (SR2012-03, Prof. Yasuhiro Kawasaki), the Japanese Ministry of Education, Culture, Sports, Science and Technology (MEXT)-Supported. Program for the Strategic Research Foundation at Private Universities (S1201022, Prof. Yasuhiro Kawasaki), and the 4th Community Medicine Promotion Grant of the Sugiura Memorial Foundation (Dr. Yukihisa Matsuda). The funders had no role in study design, data collection and analysis, decision to publish, or preparation of the manuscript.
